# Looking beyond the neonatal: what does administrative data tell us about the preschool health and developmental outcomes of children exposed to opioids in utero?

**DOI:** 10.23889/ijpds.v9i5.2582

**Published:** 2024-09-18

**Authors:** Gordon Milligan, Christian Cole, Judith Fisher, Antony Chuter, Jillian Beggs, Blair Smith, Lesley Colvin, Susan Long

**Affiliations:** 1 University of Dundee; 2 Public Member

## Objectives

To build an effective Pain data hub that benefits the public, people with chronic pain, and researchers. Alleviate is the Advanced Pain Discovery Platform (APDP) Data Hub. The aim is to make pain data Findable, Accessible, Interoperable, Reusable (FAIR) while raising awareness of chronic pain to improve the lives of people living with pain.

## Approach

Alleviate has two lived experience leads plus an Advisory Group (AG) of people with lived experience (PWLE) of chronic pain representing an online reference group of ~400 people. The leads have been involved throughout the project since inception and design, and chair regular project meetings to ensure all voices are heard equally. They lead the development of engagement materials, highlighting the importance of pain research to those with lived experience.

This co-production ensures the project to stay focussed on what matters to those living with chronic pain.

## Results

Alleviate has developed an inclusive environment for PWLE to be actively involved in decision-making and progression of the project. Together we have co-produced videos, static informational material, social media content and developed a new collaboration with an international pain charity to run a UK-wide pain survey.

## Conclusions

This co-production model has allowed us to generate impactful outputs and opportunities, raising awareness of Alleviate and pain research.

## Implications

Providing an inclusive space in the heart of an academic project for PWLE has given additional focus for researchers and provided ownership to the public in activities that directly affect them.

